# Enhanced c‐Fos expression in the central amygdala correlates with increased thigmotaxis in rats with peripheral nerve injury

**DOI:** 10.1002/ejp.839

**Published:** 2016-03-31

**Authors:** R.H. Morland, A. Novejarque, C. Spicer, T. Pheby, A.S.C. Rice

**Affiliations:** ^1^ Pain ResearchDepartment of Surgery and CancerFaculty of MedicineChelsea and Westminster Hospital campusImperial College LondonUK

## Abstract

**Background:**

Pain is associated with affective, cognitive and sensory dysfunction. Animal models can be used to observe ethologically relevant behaviours such as thigmotaxis, giving insight into how ongoing sensory abnormalities influence natural rodent behaviours. The amygdala is a complex group of nuclei implicated in the integration and generation of emotional behavioural responses, including those associated with pain, and a region known as the central amygdala is particularly associated with generation of behavioural responses, due to its links to the descending pain modulation pathways; as such, study of amygdalar c‐Fos immunoreactivity can help identify the neuronal circuits involved.

**Method:**

This study investigated changes in both nociceptive evoked responses and open field behaviour following spinal nerve transection (SNT) in male Wistar rats, and attempted to correlate these with changes in central amygdala c‐Fos immunoreactivity.

**Results:**

Fourteen days after SNT, mechanical hypersensitivity was present in the hind paw ipsilateral to site of injury. Thigmotactic behaviour was significantly increased in both SNT and sham surgery animals, with c‐Fos immunoreactivity in the central amygdala significantly greater in SNT animals compared to both sham and naive groups. Activation was greatest in the capsular and lateral subnuclei of the central amygdala, and in the caudal‐most regions. There was a strong correlation between thigmotactic behaviour and central amygdala activation following SNT surgery not seen in sham animals suggesting a role for the amygdala in behavioural responses to peripheral nerve injury.

**Conclusions:**

This study provides evidence to support the role of the amygdala in thigmotactic open field behaviour following SNT.

**What does this study add?:**

Thigmotaxis and amygdala activation are positively correlated in rats following spinal nerve transection.

Behavioural changes seen in sham animals did not correlate with amygdala activation, suggesting amygdala activation is related to nociceptive input.

Evoked measures, such as hindpaw withdrawal, are not correlated with either thigmotaxis or amygdala activation, emphasizing the importance of complex behaviours when studying pain.

## 1 Introduction

Pain involves sensory, emotional and cognitive aspects, with neuropathic pain representing a particularly complex condition arising as a direct consequence of lesion or disease of the somatosensory system (Treede et al., 2008). Evidence suggests a reciprocal relationship exists between affective disturbances and somatic symptoms (Haythornthwaite et al., 1991; Wilson et al., 2001).

In animal models, the effect of nociceptive stimuli on the affective and cognitive experience can be approximated using ethologically relevant behaviours such as burrowing (Andrews et al., 2012) and thigmotaxis (Huang et al., 2013). Behavioural alterations have been described in rat models of peripheral traumatic nerve injury (Hu et al., 2007; Blackbeard et al., 2012; Galan‐Arriero et al., 2015; Wang et al., 2015; Avila‐Martin et al., 2015; Grégoire et al., 2012), neuropathic viral infection (Hasnie et al., 2007; Wallace et al., 2007a) and drug‐induced neuropathies (Wallace et al., 2007b; Huang et al., 2013).

Evidence indicates the amygdala is a neural substrate for emotional responses to pain (Fields, 2000; Meagher et al., 2001). The lateral amygdala receives multisensory information via projections from numerous brain areas, which combined with broad inter‐connectivity across the brain enables emotional targeting of incoming stimuli (LeDoux, 2007; Martínez‐García et al., 2008; Tovote et al., 2015). The capsular and lateral regions of the central amygdala (Ce) receive purely nociceptive inputs from the dorsal horn (Gauriau and Bernard, 2002), and it is this pathway that appears to mediate unconditioned fear reactions (Borszcz and Leaton, 2003). This implicates the Ce in both modulation and generation of behavioural responses to aversive stimuli including pain (Neugebauer et al., 2004).

Functional studies corroborate the involvement of the amygdala in affective aspects of pain. In primates and non‐primate mammalian species (Borszcz and Leaton, 2003; Fudge and Tucker, 2009), lesion or inactivation of the central amygdala diminishes the expression of both “fear” (i.e. immediate response to current threat to facilitate survival) and “anxiety” (i.e. cognitive response to potential but absent threat) in response to aversive stimuli (LeDoux, 1998). Such lesions also attenuate escape‐avoidance behaviour associated with the chronic constriction model (CCI) (Pedersen et al., 2007). Clinical imaging studies have associated evoked pain with regional increases in perfusion in brain areas involved in affective‐motivational control, including the amygdala (Geha et al., 2007). Neuroimaging studies have repeatedly identified pain‐related signal changes in the amygdala, associated with mechanical allodynia in both neuropathic pain patients (Petrovic et al., 1999), and rats following CCI (Paulson et al., 2002). Furthermore, studies on c‐Fos immunoreactivity following CCI and SNL have revealed involvement of areas closely connected with the Ce including the bed nucleus of the stria terminalis (Morano et al., 2008), basolateral amygdala, and prefrontal cortex (Hayashi et al., 2009).

Thus, the Ce likely plays a key role in the generation and modulation of affective behaviours associated with peripheral nerve damage. Here, we elucidate the role of the amygdala complex in the generation of pain‐related affective alterations in an ethological behaviour in the laboratory rat, with the ultimate goal of identifying novel mechanistic targets to alleviate the suffering of patients with neuropathic pain.

## 2 Material and methods

### 2.1 Ethical statement

All animal experiments conformed to British Home Office Regulations (Animals (Scientific Procedures) Act 1986 Amendment Regulations 2012 (SI 2012/3039) under the authority of United Kingdom Home Office Project Licence 70/7162, and the International Association for the Study of Pain (IASP) guidelines for *in vivo* research (Zimmermann, 1983). Experiments were designed according to Good Laboratory Practice standards (Macleod et al., 2009) and are reported in accordance with the ARRIVE Guidelines (Kilkenny et al., 2010, https://www.nc3rs.org.uk/arrive-guidelines).

### 2.2 Experimental animals and surgical procedures

Experiments used adult male Wistar rats (Charles River, UK), weighing 240–350 g (mean 300 g) on experimental day one. The animals were housed in standard individually ventilated cages in groups of 3–4 with corncob bedding, no environmental enrichment, under a 12:12 h light/dark cycle, and temperature (25 °C) and humidity (30%)‐controlled conditions. Animals were provided with normal rat chow food (RM1 pelleted form; Special Diet Services, Essex, UK) and tap water *ad libitum* and acclimatized to their housing environment for a minimum of 48 h after arrival.

### 2.3 Study design

Necessary steps were taken to minimize the impact of experimental bias (Supplemental Table S1). The experimental unit used in this study was the individual animal – data concerning technical replicates used in immunohistochemical analyses can be found in supplementary materials (Table S2).

All experiments were performed during the light phase (08:00–18:00 hours) in our laboratories at Chelsea and Westminster Hospital campus of Imperial College. Due to the capacity of our laboratory, and to ensure comparable timings between test animals, the behavioural experiments were performed in batches (normally 3–4 animals per group). Throughout the study, the pseudorandom sequences A–B–C, B–C–A, and C–A–B were used to mask cage labels during testing. Cages were randomly allocated to experimental group by a second experimenter by picking numbers out of a hat. Allocation concealment and observer blinding of behaviour was difficult to maintain as following SNT surgery, animals show hindlimb posture changes, not observed in sham and naive rats. Cage labels were masked before surgical procedures and open field exposure. A single observer, ‘blinded’ to experimental group allocation, assessed mechanical hypersensitivity to punctate stimuli. Additionally, an independent person assigned codes to the open field videos and histological samples, only revealed following completion of data analysis. Group sizes of five (hind paw withdrawal) and eight (thigmotaxis) were determined by sample size calculations (SigmaStat, version 3.5; ANOVA sample size, desired power = 0.8, α = 0.05), with effect sizes for estimation derived from previous studies looking at the effect of spinal nerve transection on open field behaviour (Blackbeard et al., 2012).

### 2.4 Surgical procedures

Surgery was performed under general isofluorane anaesthesia (2%, Abbott, UK, O_2_ and N_2_0; 1 L/min both), and aseptic surgical conditions in a dedicated surgical laboratory. Perioperative analgesia (0.05 mL bupivacaine, AstraZeneca, UK) and antibiotic treatment [Enrofloxacin (“Baytril”): 0.2 mL/kg, Bayer Ltd, Dublin, Ireland] were injected subcutaneously at the start of the spinal nerve transection surgery, which was performed using a technique modified from Kim and Chung (Kim and Chung, 1992; Maratou et al., 2009). A 1–2 cm midline skin incision was made level with the iliac crests. The left paraspinal muscles were separated from spinous processes of L4 to S2 vertebrae using blunt dissection. Using anatomical landmarks, the L6 transverse process was identified and a small laminectomy performed, exposing L4 and L5 spinal nerve roots. The left L5 was tightly ligated (4‐0 Mersilk, Ethicon) and transected 1–2 mm distal to the ligation. Transection of the L5 nerve root was confirmed *post‐mortem* in all SNT animals and only data from animals with confirmed nerve transection were included in the analysis. The wound was sutured and animals received intraperitoneal [20% carprofen (“Rimadyl”), 0.5 mL/kg; Pfizer, Sandwich, Kent, UK] 4 h post‐surgery to provide post‐operative analgesia. Sham‐operated animals were subjected to an identical surgical procedure with the exception of the laminectomy, ligation and transection, whereas naive animals did not undergo any surgical procedure but were transported and housed in their cages in the surgical room for an equivalent period.

### 2.5 Assessment of mechanical hypersensitivity to punctate stimuli

Hind paw withdrawal to sensory stimuli was measured in conscious rats as previously described (Wallace et al., 2007a). Briefly, animals were tested in individual Plexiglas observation chambers (23 × 18 × 14 cm) by a single observer, ‘blinded’ to group allocation. The hind paw withdrawal threshold (PWT) in response to punctate static mechanical stimulation was assessed 14 days post‐injury using an electronic ‘von Frey’ device (Somedic AB, Hörby, Sweden). The calibrated force transducer (0.5 mm^2^ diameter tip) was manually applied to the mid‐plantar surface of both left and right hind paw alternately (8–15 g/s) until an active limb withdrawal response was observed (Bridges et al., 2001). The threshold value was calculated as the mean of five measurements. Two baseline values were obtained for all rats prior to surgery. Mechanical hypersensitivity was defined as a post‐operative change in the hind paw withdrawal of at least −30% from baseline – animals in the SNT group not achieving this were excluded during analysis, and reported as withdrawals (see results for details of exclusions and withdrawals).

### 2.6 Assessment of thigmotactic behaviour

The day after PWT assessment (day 15 post‐injury), rats were introduced into the near left corner of a square, black open field arena (100 × 100 cm with a virtual inner zone of 40 × 40 cm) under dim light (12 lux, LED). The open field was enclosed in an isolation chamber to minimize environmental disruption. Spontaneous locomotor behaviour of the rats was recorded for a total of 15 min (Hasnie et al., 2007; Wallace et al., 2007a) using a high sensitivity camera (VCB 3372; Sanyo, Moriguchi, Osaka, Japan). The arena was cleaned with 0.02% Distel (formerly Trigene; Tristel Solutions Ltd., Snailwell, Cambridgeshire, UK) between trials. Following exposure to the open field arena, animals were housed individually, under the same conditions to minimize exposure to new stimuli, for 90 min prior to perfusion. The video files captured during each trial were analysed using Ethovision software XT 4.1.106 (Tracksys, Nottingham, UK; for Noldus, The Netherlands), with frequency of entry into the inner zone as the primary outcome measure. Secondary measures recorded were total distance travelled, distance travelled in the inner zone, duration in the inner zone, and rearing behaviour. Rearing was defined as both forelimbs elevated, both wall‐supported and freestanding, and measured by a single‐trained observer, blinded to group allocation, watching recorded videos at 4× playback speed.

### 2.7 Immunohistochemistry

Ninety minutes after open field exposure, animals were humanely killed with an intraperitoneal overdose of sodium pentobarbital (0.65 mL of Euthatal; Merial Animal Health Ltd, Harlow, Essex, UK) and transcardially perfused with saline (0.9% NaCl) followed by fixative (4% paraformaldehyde in 0.1 M phosphate buffer; PB, pH 7.6; Sigma‐Aldrich Ltd, Gillingham, Dorset, UK). Brains were dissected and right hemisphere marked with a longitudinal incision for future identification, before being post‐fixed (4 h) in the same fixative and immersed in 30% sucrose solution in 0.1 M PB until they sank. Ten parallel series of frontal sections (50 μm) were obtained using a freezing microtome. Endogenous peroxidase was inhibited with 0.003% H_2_O_2_ (Sigma‐Aldrich) in 0.1 M saline phosphate buffer (PBS) for 30 min at room temperature (RT; 20 °C), followed by 1 h blocking in 5% normal goat serum [NGS, Millipore, UK; PBS with 0.3% TX (Triton X‐100, BDH, UK)]. Sections were then washed in PBS and incubated overnight in rabbit IgG anti‐c‐Fos (1:20.000, Santa Cruz Biotechnology, Santa Cruz, CA, USA) in PBS containing 0.3% Triton X‐100 and 2% normal goat serum (NGS) at 4 °C. After being washed in PBS, sections were incubated with goat anti‐rabbit IgG biotinylated (1:250; Jackson ImmunoResearch Laboratories, Westgrove, PA, USA) in PBS containing 0.3% Triton X‐100 and 2% NGS for 2 h (RT). Sections were PBS washed prior to incubation with avidin–biotin complex (ABC Elite kit; Vector Laboratories, Burlingame, CA, USA) in PBS with 0.3% Triton X‐100 for 90 min (RT). After two PBS washes and one PB wash, peroxidase activity was revealed via 3,3′‐diaminobenzidine visualization (DAB, Vector Laboratories Ltd, Peterborough, UK) with nickel salt intensification (DAB‐Ni). Sections were incubated for 30 min and the reaction was stopped with PB and PBS washes. Sections were rinsed in warm (37 °C) 0.2% gelatine (Gelatine Type A, from Porcine skin; Sigma‐Aldrich Ltd) in PB and mounted on clean slides, counterstained with acidic toluidine blue and cover‐slipped with DePex (VWR International Ltd, Lutterworth, Leicestershire, UK) for analysis.

### 2.8 Image analysis and quantification

The slides were coded to maintain blinding to tissue treatment conditions. Cells labelled with the c‐Fos immunohistochemistry were counted using published guidelines (Burke et al., 2003, 2013). Counts were obtained for both hemispheres from all sections containing the central amygdala, as defined in the stereotactic atlas of Paxinos and Watson (2007); antero‐posterior coordinates covering Bregma −1.44 mm and −3.36 mm). Slides were digitally captured using a 10× objective and Leica DMR fluorescence microscope (Leica, Milton Keynes, UK) with a Hamamatsu CCD camera (C5810 CCD camera; Hamamatsu Photonics Ltd, Welwyn Garden City, Hertfordshire, UK) using Leica QWin v.3.0 software (Leica Microsystems Ltd, Milton Keynes, Buckinghamshire, UK) for optic imaging (with consistent exposure times). Digital images were imported and stitched using Adobe Photoshop CS5 (Adobe Systems, Mountain View, CA, USA). The cytoarchitecture of the central amygdaloid subnuclei was delineated using spatial landmarks and reference to Paxinos and Watson (Paxinos and Watson, 2007), and the area of each subnuclei measured. The blue channel was eliminated to facilitate the selection of the labelled neurons. No additional filtering or manipulation of the images was performed. The criterion for c‐Fos‐labelled cells was a uniform black reaction product within the cell nucleus and was confirmed by observing the slides at high magnification. In each section, c‐Fos‐labelled cells were counted using Adobe Photoshop CS5. All results represented the density of labelling as number of cells/mm^2^ and the final value was expressed as mean (SEM) of the labelled cell density. The resultant densities were presented in three antero‐posterior levels of central amygdala and each of its subnuclei. Multiple sections were pooled within each animal to produce a mean value. The number of sections analysed for naive, sham, and SNT animals are detailed in Table S2.

### 2.9 Statistical analysis

Sigmastat version 3.5 (SPSS Inc., Surrey, UK) was used to test for statistically significant differences (*p* < 0.05) throughout the study. For mechanical hypersensitivity measures, groups were compared using two‐way repeated measures ANOVA, looking at time effects for each paw, and laterality effects for each time point. Thigmotactic behaviour measures were compared using one‐way ANOVA with the appropriate *post hoc* analysis (Holm–Sidak method, Tukey test when the equal variance test failed, and Dunn's method where the data were not normally distributed, with appropriate multiple comparison corrections applied). To explore the effect of the treatment in the antero‐posterior expression of c‐Fos in the Ce and its subnuclei, a three‐way ANOVA was used, with group (naive, sham, and SNT) as between‐subjects factor and rostro‐caudal level of the central amygdala and subnuclei as within‐subjects factors. Comparison of c‐Fos immunoreactivity in both hemispheres was conducted using two‐way ANOVA. Finally, the presence of correlations between the mechanical hypersensitivity, open field behaviour, and Ce c‐Fos expression were investigated using Pearson's Correlation. Data are reported as mean values ± standard error of the mean (SEM).

## 3 Results

**Figure 1:**
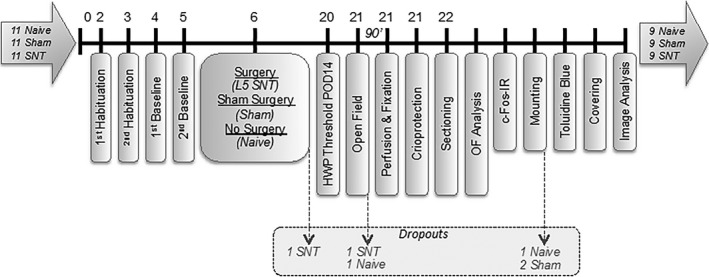
Experimental design and dropouts. One rat died during recovery time following L5 spinal nerve transection (SNT) surgery. Another SNT animal was excluded due to changes in the lighting conditions during the open field test. One naive animal also performed in the open field arena outside of the protocol conditions during the paradigm. The right hemispheres of one naive and two sham rats could not be identified and therefore these brain frontal sections were not analysed.

One SNT animal died following surgery and was excluded from the analysis (see Fig. 1). All rats in the SNT group showed a post‐operative change in hind paw withdrawal of at least 30% from baseline and were included according to *a priori* exclusion criteria. Furthermore, two animals were excluded due to breaches in environmental conditions during the open field paradigm (one SNT due to lighting fluctuations; one naive due to olfactory disturbances). During immunohistochemical analysis, there were three exclusions due to a lack of hemispheric identification (one naive, two sham).

### 3.1 Development of mechanical hypersensitivity following spinal nerve transection

**Figure 2:**
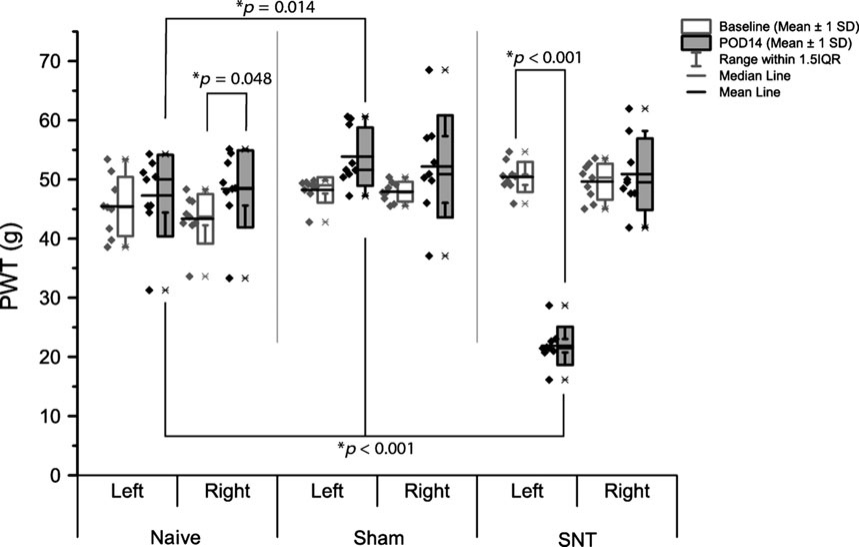
Mechanical hypersensitivity induced by L5 SNT. Ipsilateral (left) and contralateral (right) hind paw withdrawal thresholds (PWT) in response to electronic von Frey mechanical stimulation. Differences between and within groups were determined by two‐way repeated measure ANOVA. There were no differences in contralateral PWT, in contrast with ipsilateral data, which revealed a marked decrease in threshold in SNT animals 14 days after surgery. Mean and median are given to aid in data interpretation, highlighting variations in data distribution between groups and outcomes (*n* = 9).

Hind PWT in response to punctate mechanical stimulation were measured with an electronic von Frey device at baseline and 14 days post‐surgery (Fig. 2).

Two‐way repeated measure ANOVA on ipsilateral PWTs revealed a significant effect of time point (*F*(8,1) = 46.16, *p* = 0.0001) and group (*F*(16,2) = 51.27, *p* < 0.0001). A significant interaction between group and timepoint was also detected (*F*(16,2) = 68.08, *p* < 0.0001, Holm‐Sidak post hoc test *p* < 0.001), which further post‐hoc analyses identified as due to significant differences between groups at POD (post‐operative day) 14. No significant effects were seen in baseline outcomes. Analysis of data from the contralateral/right paw revealed no significant group effect (*F*(16,2) = 2.91, *p* = 0.84), but a significant time point effect (*F*(8,1) = 6.62, *p* = 0.033, Holm–Sidak *post hoc* test *p* < 0.001), with *post hoc* analysis revealing a small increase in naive PWT between baseline and POD 14 (Holm–Sidak *post hoc* test *p* = 0.048).

The decrease in PWT was not associated with overt motor deficit, as confirmed by locomotor activity in the open field reported below.

### 3.2 Spinal nerve transection and sham surgery increase thigmotaxis

**Figure 3:**
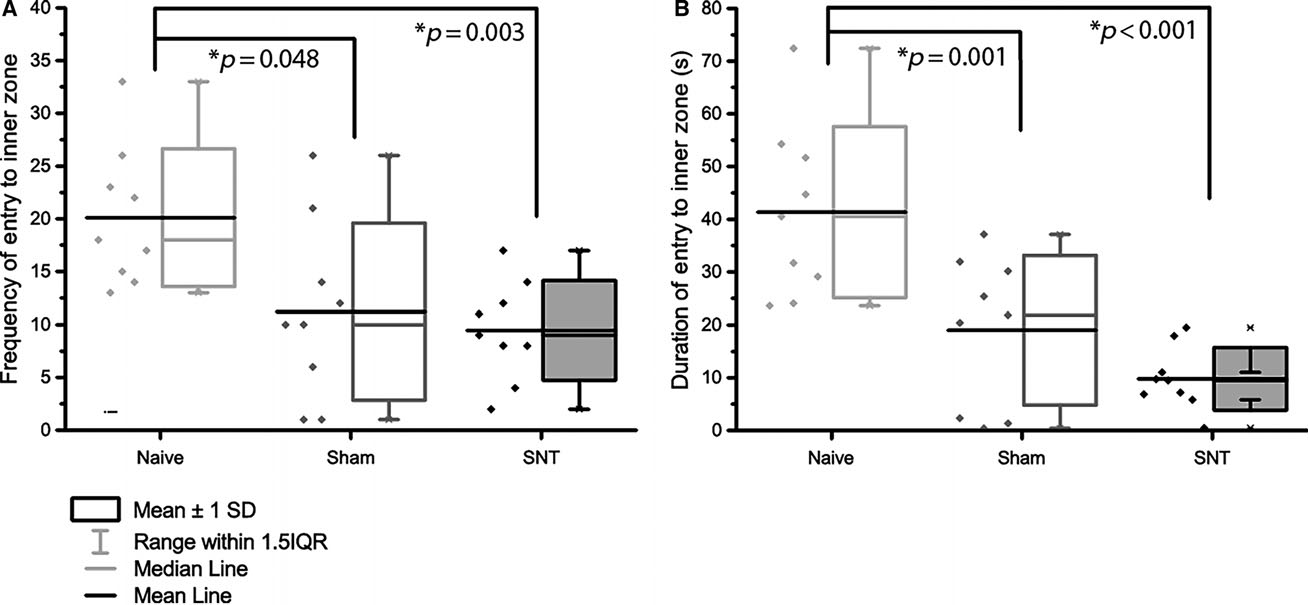
Thigmotactic behaviour in the open field following L5 SNT. Exploration in the open field was assessed 15 days after L5 SNT surgery. (A) The number of entries into the inner zone was significantly reduced in both SNT (*p* = 0.0025) and sham (*p* = 0.0096) surgery compared to naive animals. (B) The duration spent in the inner zone was also significantly decreased in both SNT (*p* < 0.001) and sham (*p* = 0.0012) compared to naive animals. Data were analysed using one‐way ANOVA and Sidak–Holm *post hoc* test. Significance taken at *p* < 0.05 (*n* = 9).

**Figure 4:**
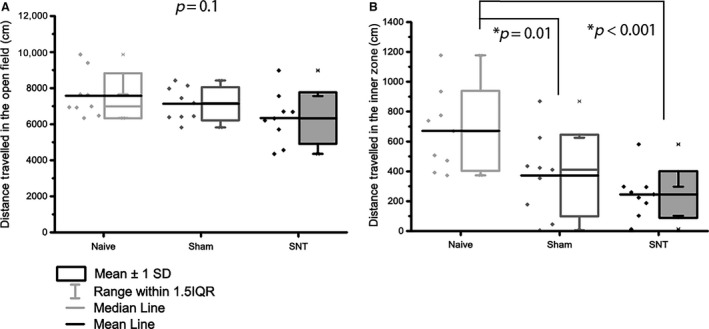
Locomotor activity in the open field following L5 SNT. Locomotion is not impaired in rats following L5 SNT. (A) The total distance travelled in the open field was not significantly altered 15 days after surgery. (B) Distance travelled in the inner zone was significantly decreased in both SNT (*p* < 0.001) and sham (*p* = 0.014) following surgery. Data were analysed using one‐way ANOVA and Sidak–Holm *post hoc* test. Significance taken at *p* < 0.005 (*n* = 9).

Having demonstrated development of hind paw mechanical hypersensitivity in SNT rats at POD 14, we used an open field arena (OF) to assess possible alterations in spontaneous exploratory activity previously observed in rodent models of traumatic nerve injury (Wallace et al., 2007b; Blackbeard et al., 2012; Huang et al., 2013). There was a significant effect of group on both duration in the inner zone (*F*(24,2) = 14.25, *p* < 0.001), and frequency of entry (Kruskal–Wallis chi‐squared value = 10.427, df = 2, *p* = 0.0054). SNT animals displayed increased thigmotactic behaviour (Fig. 3) reflected by a significant decrease in frequency of entry to (9.44 SEM 1.56), and time spent in (4.76 s SEM 0.98 s) the inner zone compared to naive animals (18.70 SEM 2.4 and 23.05 s SEM 5.09 s respectively; Kruskal–Wallis one‐way ANOVA with Tukey *post hoc* test, *p* = 0.002; one‐way ANOVA with Holm–Sidak *post hoc* test, *p* = 0.005). This decrease in frequency of inner zone entry was also observed in sham animals versus naive (11.22 ± 2.79; one‐way ANOVA with Holm–Sidak *post hoc* test, *p* = 0.005), with no significant difference detected between sham and SNT animals. There was no significant difference in the total distance moved (Fig. 4A, naive = 7505.42 ± 379.78 cm; sham = 7136.79 ± 306.51 cm; SNT = 6342.27 ± 474.68 cm; *F*(24,2) = 2.39, *p* = 0.11), indicating that the surgery did not impair locomotion. However, the distance covered in the inner zone by both SNT and sham animals was significantly reduced compared to naive (one‐way ANOVA *F*(25,2) = 7.55, *p* = 0.0029, Tukey *post hoc p* < 0.001, *p* = 0.014 SNT and sham respectively; Fig. 4B) denoting avoidance of the inner zone. No significant difference was seen in rearing behaviour revealed a significant overall difference (Kruskal–Wallis chi‐squared value = 5.07, *p* = 0.079; naive = 84.10 SEM 6.39; sham = 84.56 SEM 6.41; SNT = 61.33 SEM 8.02).

### 3.3 Spinal nerve transection and central amygdaloid c‐Fos immunoreactivity after exposure to the open field

**Figure 5:**
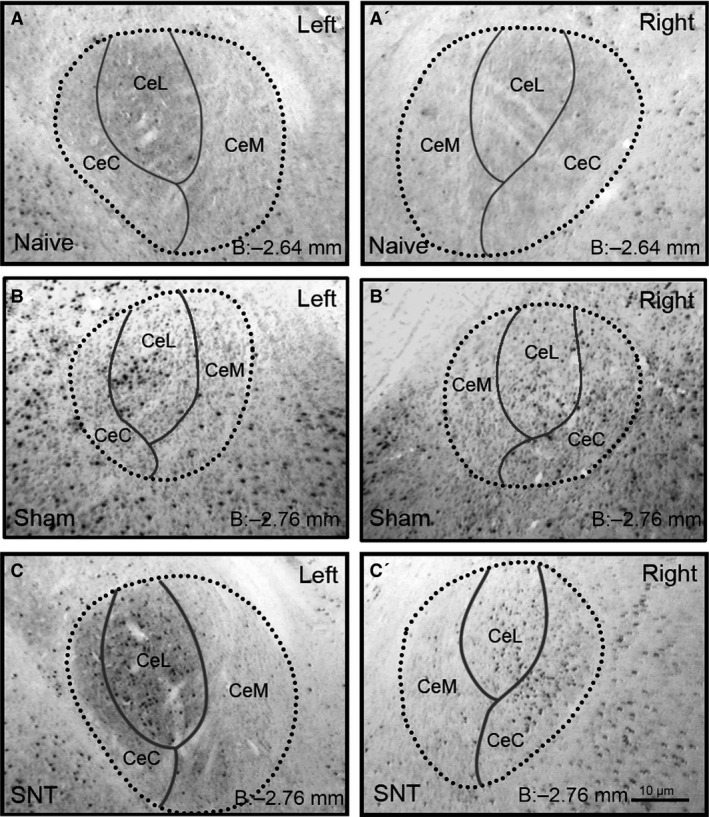
Examples of c‐Fos immunoreactivity. Illustrating the staining observed and subdivision of nuclei, comparing left and right hemispheres across the three experimental groups. Dark round puncta indicative of c‐Fos immunoreactivity are clearly visible, as are cytoarchitectural features such as the external capsule used to delineate the central amygdala and its subnuclei. Bregma coordinates as stated in lower right corner of each image correspond to the intermediate rostro‐caudal level overall, and for CEL/CEC, with CeM level corresponding with the caudal region of the subnuclei.

**Figure 6:**
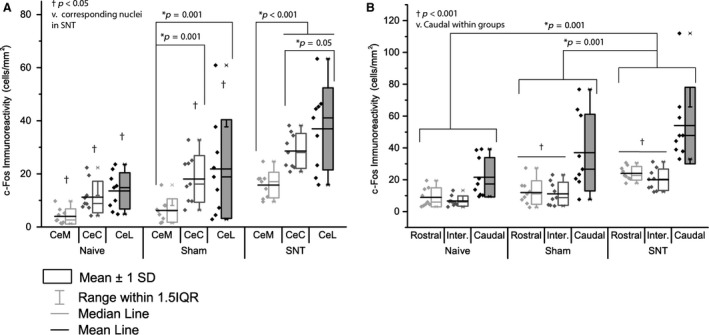
c‐Fos immunoreactivity is increased in the Ce following L5 SNT surgery. A) Global activation of c‐Fos in the central amygdala was increased in the SNT group compared to both naive (*p* < 0.001) and sham (*p* = 0.0048). B) No lateralization was observed, with left and right hemispheres exhibiting similar levels of immunoreactivity. *n* = 9 (biological replicates), *n* = 6–10 (technical replicates; number of sections averaged for each animal, with full details in Table S2).

As c‐Fos immunoreactivity peaks 90 min after stimuli (Hunt et al., 1987), animals were housed individually between open field exposure and perfusion to minimize further stimulation. Their brains were then processed for immunohistochemical analysis of c‐Fos expression in the central amygdala (Ce) and its subnuclei in both hemispheres (Fig. 5). The combined study of thigmotactic behaviour and c‐Fos expression in the Ce in response to exposure to the OF allows us to analyse whether these structures show changes associated with this mild threat‐environment stimulus. It also enables investigation into how such changes differ depending on experimental treatment, and determine the effect of spinal nerve injury on Ce function in response to the exploration of the OF. Examples of c‐Fos immunoreactivity from both hemispheres of all three experimental groups can be seen in Fig. 6, with data summarized in Table S3.

**Figure 7:**
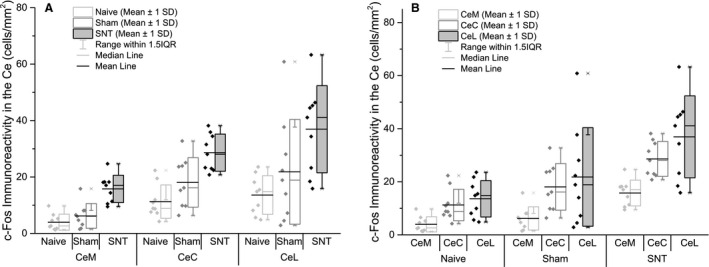
Subnuclei of the Ce show differential patterns of c‐Fos immunoreactivity in response to open field exposure. (A) c‐Fos immunoreactivity was significantly lower in the CeM compared to the CeL and CeC (*p* < 0.001). (B) Following L5 SNT, c‐Fos immunoreactivity in all subnuclei was increased compared to naive and sham (*p* < 0.001), in addition to higher levels being noted in sham compared to naive (*p* = 0.032). Data were analysed using two‐way ANOVA, with significance taken at *p* < 0.05. *n* = 9 (biological replicates), *n* = 6–10 (technical replicates; number of sections averaged for each animal, with full details in Table S2).

A three‐way ANOVA was conducted to investigate the effect of L5 SNT surgery on c‐Fos immunoreactivity in the subnuclei, and along the rostro‐caudal gradient within the Ce. Group allocation (*F*(2) = 38.3, *p* < 0.001), subnuclei (*F*(3) = 18.16, *p* < 0.001) and rostro‐caudal level (*F*(2) = 26.01, *p* < 0.001) contributed significantly to the patterns observed. An interaction was present between subnuclei and rostro‐caudal level (*F*(5) = 7.55, *p* < 0.001), but no interaction was detected between group allocation and either level (*F*(4) = 1.45, *p* = 0.22) or subnuclei (*F*(6) = 1.24, *p* = 0.29). Fig. 7 shows the pattern of c‐Fos immunoreactivity between subnuclei and along the rostro‐caudal gradient.

Holm–Sidak *post hoc* multiple comparisons were performed to identify areas of the Ce that show differential activation following exposure to the open field. At the group level, SNT animals showed higher levels of c‐Fos immunoreactivity versus both naive and sham groups (*p* < 0.001), with sham animals also exhibiting significantly increased activation compared to naive (*p* = 0.027), as shown in Fig. 5A.

c‐Fos immunoreactivity in caudal amygdalar regions was significantly higher than that seen in either intermediate or rostral regions (*p* < 0.001; no significant difference between groups, *p* = 0.61), as shown in Fig. 7B. Looking at interactions within rostro‐caudal levels, naive animals showed no significant differences, whereas both SNT and sham animals displayed significant up‐regulation in caudal regions compared to both intermediate (sham *p* = 0.013, SNT *p* < 0.001) and rostral (*p* = 0.015, *p* = 0.001; as previous). Looking at group differences at each level, c‐Fos immunoreactivity was consistently higher in the SNT group as compared to both sham (rostral *p* = 0.005, intermediate *p* = 0.02, caudal *p* < 0.001) and naive (*p* < 0.001, *p* = 0.001, *p* < 0.001 as above).

No evidence of lateralization was observed following open field exposure, as shown in Fig. 5B (three‐way ANOVA *F*(1) = 0.02, *p* = 0.89).

Holm–Sidak *post hoc* multiple comparisons within groups revealed no differences between subnuclei in naive animals. Sham animals showed significantly lower activation in the CeM compared to the CeL (*p* = 0.001) and CeC (*p* = 0.001), with no difference between CeC and CeL activation (*p* = 1). Following SNT, significantly lower CeM activation was noted in comparison with CeC (*p* < 0.001) and CeL (*p* < 0.001), with a further significant difference observed between CeC and CeL, with the CeL showing the highest overall levels of activation (*p* = 0.047). As would be expected, comparison of group within subnuclei revealed greatest activation in the SNT group, with all nuclei showing a significant difference between SNT and sham (CeM *p* = 0.009, CeC *p* < 0.001, CeL *p* < 0.001), and a difference between sham and SNT animals noted in the CeC (*p* = 0.018) and CeL (*p* < 0.001), but not the CeM (*p* = 0.027). Fig. 7A summarizes the c‐Fos immunoreactivity across the three subnuclei according to experimental group.

Analysis of the interaction between subnuclei and rostro‐caudal gradient revealed no significant differences between nuclei at the rostral level (*p* = 0.23, *p* = 0.47, *p* = 64, CeM vs. CeC, CeM vs. CeL, and CeC vs. CeL, respectively). At the intermediate level, CeL showed significantly higher levels of immunoreactivity compared to the CeM (*p* = 0.001), with no other differences noted. At the caudal level, CeL and CeC showed significantly higher c‐Fos immunoreactivity compared to the CeM (*p* < 0.001 for both). Looking at the inverse relationship, there were no differences in CeM activation related to rostro‐caudal level (*p* = 0.9, *p* = 0.16, and *p* = 0.76 for rostral vs. caudal, rostral vs. intermediate, and intermediate vs. caudal). In both the CeC and CeL, c‐Fos immunoreactivity was significantly higher in the caudal region compared to both intermediate and rostral levels (*p* < 0.001 for all).

### 3.4 Correlations between behavioural and immunohistochemical outcomes

Finally, an analysis of correlations between all outcome measures across the three experimental groups was performed (Table 1). Across all groups, calculation of the Pearson's correlation coefficient indicated significant positive correlations between number of entries into and duration in the inner zone. Significant positive correlations were found between the Ce activation and the activation of its subnuclei, indicating that each of the Ce subdivisions contribute to the global activation of the Ce.

**Table 1:** (A) Correlations between open field behaviour and hind paw hypersensitivity. No correlations between POD 14 PWT and open field were observed. Data represent Pearson correlation coefficients, with ρ stated, and significance taken at *p* < 0.05, as denoted by *. (B) Correlations between open field behaviour and c‐Fos activation in the central amygdala. Differences in correlations are seen between groups, with SNT animals exhibiting the highest correlation between open field behaviour and c‐Fos immunoreactivity in the Ce. Data represent Pearson correlation coefficients, with ρ stated, and significance taken at *p* < 0.05, as denoted by *

(A)		Naive					Sham					SNT			
		Dur.	Freq.	Total Dist.	IZ Dist.	Rears	Dur.	Freq.	Total Dist.	IZ Dist.	Rears	Dur.	Freq.	Total Dist.	IZ Dist.
Frequency Total Dist. (cm)	ρ	0.78					0.89					0.91			
	Sig.	0.01*					0.00*					0.00*			
	ρ	0.40	0.52				0.80	0.77				0.47	0.69		
Inner Zone Dist. (cm)	Sig.	0.29	0.15				0.01*	0.01*				0.21	0.04*		
	ρ	0.90	0.90	0.58			0.77	0.92	0.55			0.83	0.83	0.68	
	Sig.	0.00*	0.00*	0.10			0.02*	0.00*	0.12			0.01*	0.01*	0.04*	
Rearing	ρ	0.33	0.80	0.53	0.55		0.05	−0.11	−0.13	−0.20		0.67	0.83	0.57	0.45
	Sig.	0.38	0.01*	0.14	0.12		0.90	0.78	0.73	0.61		0.05	0.01*	0.11	0.22
Baseline PWT (g)	ρ	0.35	0.05	−0.33	0.08	−0.22	−0.63	−0.83	−0.58	−0.88	0.00	−0.10	−0.08	−0.12	0.05
	Sig.	0.36	0.90	0.38	0.83	0.58	0.07	0.01*	0.10	0.00*	1.00	0.80	0.85	0.77	0.90
POD 14 PWT (g)	ρ	−0.15	0.12	−0.20	−0.12	0.38	−0.10	−0.12	−0.42	0.00	−0.47	0.38	0.47	−0.07	0.18
	Sig.	0.70	0.77	0.61	0.77	0.31	0.80	0.76	0.26	1.00	0.21	0.31	0.20	0.86	0.64
(B)															
CeA	ρ	0.10	0.42	−0.32	0.10	0.60	−0.05	−0.21	−0.23	−0.08	−0.13	−0.73	−0.77	−0.70	−0.97
	Sig.	0.80	0.26	0.41	0.80	0.09	0.90	0.59	0.55	0.83	0.73	0.02*	0.02*	0.04*	0.00*
Left CeA	ρ	0.18	0.48	−0.27	0.15	0.62	0.05	−0.12	−0.15	−0.02	−0.07	−0.30	−0.41	−0.35	−0.60
	Sig.	0.64	0.19	0.49	0.70	0.08	0.90	0.76	0.70	0.97	0.86	0.43	0.27	0.36	0.09
Right CeA	ρ	0.17	0.23	−0.27	0.18	0.25	0.00	−0.11	−0.12	0.03	−0.18	−0.77	−0.78	−0.72	−0.93
	Sig.	0.67	0.55	0.49	0.64	0.52	1.00	0.78	0.77	0.93	0.64	0.02*	0.01*	0.03*	0.00*
CeM	ρ	−0.07	0.22	−0.17	−0.17	0.47	−0.13	−0.16	−0.23	−0.02	−0.28	−0.83	−0.61	−0.23	−0.78
	Sig.	0.86	0.58	0.67	0.67	0.21	0.73	0.68	0.55	0.97	0.46	0.01*	0.08	0.55	0.01*
CeC	ρ	−0.12	−0.02	−0.48	−0.23	0.25	−0.17	−0.27	−0.22	−0.07	−0.08	−0.73	−0.77	−0.67	−0.97
	Sig.	0.77	0.97	0.19	0.55	0.52	0.67	0.48	0.58	0.86	0.83	0.02*	0.02*	0.05	0.00*
CeL	ρ	0.50	0.72	−0.10	0.52	0.60	0.10	−0.02	−0.20	0.07	0.02	−0.65	−0.74	−0.53	−0.77
	Sig.	0.17	0.03*	0.80	0.15	0.09	0.80	0.97	0.61	0.86	0.97	0.06	0.02*	0.14	0.02*
Rostral CeA	ρ	−0.17	0.08	−0.40	−0.27	0.37	−0.22	−0.17	0.03	−0.15	−0.17	−0.92	−0.85	−0.32	−0.80
	Sig.	0.67	0.83	0.29	0.49	0.33	0.58	0.67	0.93	0.70	0.67	0.00	0.00*	0.41	0.01*
Intermediate CeA	ρ	0.02	0.50	0.13	0.15	0.80	−0.18	−0.25	−0.23	−0.08	−0.20	−0.73	−0.67	−0.38	−0.87
	Sig.	0.97	0.17	0.73	0.70	0.01*	0.64	0.51	0.55	0.83	0.61	0.02	0.05	0.31	0.00*
Caudal CeA	ρ	0.03	0.32	−0.13	−0.02	0.53	0.33	0.13	−0.07	0.30	0.15	−0.10	−0.15	−0.28	−0.30
	Sig.	0.93	0.41	0.73	0.97	0.14	0.38	0.75	0.86	0.43	0.70	0.80	0.70	0.46	0.43

Ce, central nucleus of the amygdala; CeC, capsular subnuclei of the central amygdala; CeL, lateral subnuclei of the central amygdala; CeM, medial subnuclei of the central amygdala; PWT, hind paw withdrawal threshold; SNT, spinal nerve transection; POD, post‐operative day.

Looking at differences between correlation profiles of the three experimental groups, SNT was associated with a strong negative correlation between inner zone activity (duration, frequency, distance and velocity) and c‐Fos activation within the amygdala. This translates to high c‐Fos activation correlating positively with thigmotactic behaviour (avoidance of the exposed inner zone).

A strong negative correlation between open field behaviour and c‐Fos immunoreactivity in the Ce was seen in SNT animals, which showed a relationship between both duration and distance in the inner zone, emphasizing the positive relationship between thigmotactic behaviour and c‐Fos immunoreactivity in the Ce.

## 4 Discussion

L5 spinal nerve transection (SNT) is associated with mechanical hypersensitivity and increased c‐Fos expression in the central amygdala (Ce) following open field exposure. Both SNT and sham animals showed thigmotactic behaviour in the open field, but it only correlated with central amygdalar c‐Fos immunoreactivity in SNT animals, particularly within the capsular and lateral subnuclei, and caudal regions.

Fourteen days after L5 SNT, hypersensitivity was noted in the left/ipsilateral hind paw. No sensory changes were detected in the right/contralateral paw of SNT animals, or in either paw of naive and sham animals. Changes were not associated with a reduction in locomotion, as evidenced by constant locomotor activity, which was within the 6000–8000 cm range seen in previous studies (Morland et al., 2015; Hasnie et al., 2007; Wallace et al., 2007b; Blackbeard et al., 2012). Previous studies have shown that hind paw mechanical withdrawal thresholds (PWT) are reduced following L5 and L5/L6 SNT, with hypersensitivity evident from 2 to 4 days (Lee and Kim, 2007; Blackbeard et al., 2012) to 10 weeks, returning to baseline by 14 weeks post‐surgery (Leinders et al., 2013). Interestingly, a small increase in contralateral PWT was noted in naive animals between baseline and post‐operative day 14. This could be an effect of habituation to the PWT testing protocol, and may have been ameliorated by surgical effects in the SNT and sham animals.

Open field analysis revealed a thigmotactic phenotype of animals in the L5 SNT and sham groups, as evidenced by reduced inner zone activity (frequency and duration of entry, and distance covered in the inner zone). This suggests that sham surgery induces an intermediate behavioural phenotype: Lacking evoked hypersensitivity, but exhibiting behavioural disturbances comparable to SNT. To elucidate factors that contribute to the sham phenotype, it would be worthwhile to determine whether evoked hypersensitivity is present at the site of surgery (i.e. the lower back), as the presence of reduced thresholds in this area would indicate nociceptive signals linked to affective alterations in sham animals, highlighting the importance of comparison with naive data.

Studies into thigmotaxis have been conducted in this laboratory for the last 10 years. Previous data have shown 14 days after SNT, animals show increased thigmotaxis and decreased locomotor activity, ameliorated by gabapentin treatment (Hasnie et al., 2007). This paper also investigated the thigmotactic effect of varicella zoster infection and partial sciatic nerve injury (PSNI), noting VZV was the only model associated with thigmotactic effects without locomotor depression, with PSNI resulting in a similar behavioural phenotype to SNT, minus the response to gabapentin. Other studies from this group found inner zone duration was reduced 7 days after SNT, and noted reduced distance travelled, indicative of motor impairment when compared to naive animals (Blackbeard et al., 2012), with a later study on PSNI also demonstrating an association with increased thigmotactic behaviour in rats compared to sham animals (Wallace et al., 2007c). Thigmotactic deficits are sensitive to pharmacological intervention, as demonstrated by studies showing gabapentin increases inner zone activity to naive levels in SNT (Hasnie et al., 2007), and also in ddC‐treated animals (Wallace et al., 2008; Smith et al., 2014), with similar effects seen in morphine or diazepam treatment of ddC‐induced neuropathy (Wallace et al., 2008), whereas the microglial inhibitor, minocycline, only ameliorates thigmotactic deficits in animals treated with ddC combined with GP‐120 HIV viral protein (Wallace et al., 2007b). Previous data on open field locomotor effects suggest that both traumatic and inflammatory (Morland et al., 2015) pain models are associated with decreased locomotor activity at the time points tested, whereas systemic induction methods such as viral and drug‐induced models are not. The present study tested open field behaviour at a later time point (day 15), and found no reduction in distance travelled, suggesting the presence of a post‐surgical effect, resolved by day 14. It is also possible that the reduction in ambulation detected at the earlier time point represents a combination of motor impairment and general post‐surgical malaise, although published sham data on the subject are lacking.

Significant increases in Ce c‐Fos activation were seen in the L5 SNT group, with sham animals differing from both SNT and naive at the overall level only, with increases seen at the global level, in all subnuclei, and in the caudal and intermediate regions. Lateralization of effect was also assessed and although no overall effect was seen, it is noted that more correlations between open field behaviour and amygdala activation involved the right CeA over the left CeA. This contrasts with electrophysiological data suggesting the lateralization of amygdala activation following spinal nerve ligation (SNL) is time dependent, with left activation occurring in the acute phase (2–6 days post‐injury), and right activation predominating after this point (Gonçalves and Dickenson, 2012). However, lateralization was primarily detected in response to evoked stimuli, with comparable levels of spontaneous firing in both hemispheres. As we were looking at patterns of c‐Fos immunoreactivity associated with exposure to a mildly threatening environment, the lack of lateralization detected suggests a link between lateralization and somatic expression of behaviour, and may be associated primarily with evoked nociception, rather than the measurement of subtle changes in ethologically relevant behaviours associated with an ongoing, persistent and spontaneous pain state, such as burrowing and open field.

Amygdalar neuronal activation has been previously documented in association with peripheral nerve injury – e.g. increased blood flow and cell proliferation was observed in the Ce following spared nerve injury (SNI) (Gonçalves et al., 2008), although this was not accompanied by altered elevated plus maze or open field behaviour. In a second study, SNL increased thigmotaxis and was associated with a concomitant increase in amygdalar expression of pro‐inflammatory cytokine IL‐10 (Burke et al., 2013). Activation in sham animals was only significantly different from naive animals at the overall level, highlighting the experiential difference between SNT and sham.

Activation of the amygdala in response to experimental pain states has been previously demonstrated, both under neuropathic (Rouwette et al., 2011) and visceral pain conditions (Araki et al., 2014; De Lange et al., n.d.).

The medial Ce (CeM) showed the lowest activation in common with previous studies into the distribution of activation between amygdala subnuclei (Morland et al., 2015). The capsular and lateral subnuclei contained the highest number of positively stained cells in all animals, with SNT animals showing significantly higher levels than all other groups, consistent with the hypothesized function of these subnuclei in modulation of incoming noxious signals.

Correlation analyses revealed no link between PWTs and either Ce c‐Fos activation or open field thigmotactic behaviour. In contrast, a strong correlation was seen in SNT animals between thigmotactic outcomes and Ce activation, with high activation correlating with avoidance of the inner zone. This emphasizes the complexity of the pain experience by suggesting the link between behaviour and sensory alterations is not direct. A study into SNI‐associated behaviours showed expression of “affective” behavioural changes have an onset‐lag of up to 4 months compared to sensory symptoms (Seminowicz et al., 2009), with a second finding reduced locomotor and rearing activity 1 month after SNT or associated sham surgery (Kontinen et al., 1999). Furthermore, differences in the behavioural response to nerve injury are age‐dependent (Leite‐Almeida et al., 2009), and may be associated with the laterality of the original injury (Leite‐Almeida et al., 2012) suggesting disparity in the literature may be related to protocol variations.

Increased activation in caudal Ce regions has been previously documented in association with visceral pain (Bon et al., 1998; Morland et al., 2015), and this current study indicates that these increases are not related to a specific model of experimental pain, but are representative of a higher state of arousal. This could be determined by conducting further studies looking at the rostro‐caudal activation gradient in rats under varying levels of paradigm familiarity, hypothesizing that caudal activation is related to either increased arousal associated with novel environment or the “unpredictable inner environment” associated with spontaneous pain states. Further studies, including a third group of animals without open field exposure, would provide insight into whether the changes observed, particularly those involving caudal activation, are associated with open field exposure or merely an unrelated background process. Additionally, investigation of extra‐amygdala areas, such as the hippocampus and insula would enable greater understanding of the neural circuits underlying behavioural changes observed. This last point is particularly important as although strong correlations were observed in SNT animals, there were also some positive correlations present in sham and naive animals, suggesting the amygdala is only responsible for part of the behaviour observed, with contributions coming from other brain areas, such as those mentioned above.

The data presented here reiterate the established development of mechanical hypersensitivity following SNT, and in combination with other data on thigmotactic phenotype, it is apparent that nociceptive behavioural phenotypes vary over time in relation to underlying mechanisms. Ethologically relevant behavioural outcomes take longer to manifest in comparison with evoked sensory measures, as illustrated by the differences in open field ambulation at 7 and 14 days post‐SNT (Blackbeard et al., 2012). A time point analysis of open field behaviour at various points following nerve injury would be instructive in terms of identifying the “plateau point” and duration of thigmotactic behaviour and would allow further study into how such outcomes are linked (i.e. is there a consistent lag between expression of sensory dysfunction and behavioural abnormalities?). As ‘sham’ surgery is associated with thigmotactic alterations without a correlated increase in amygdalar activation, it is clear that detecting thigmotaxis alone is not necessarily evidence of an affectively disruptive nociceptive state. Regarding the sham group, it is important to note that measurements were taken 14 days after surgery, and it is therefore possible that some surgical effects were still present. It would be worthwhile to repeat this study, examining behaviour at a later time point to determine the contribution of general surgical effects to the behavioural changes observed. In addition, post‐how power calculations highlight this study is underpowered to detect changes in rearing behaviour, so additional studies to address this would be informative (See supplementary materials for details of *post hoc* power calculations).

This study supports the hypothesis that increases in Ce activation are associated with negative behavioural responses to peripheral nerve injury, such as thigmotaxis, and highlights the disconnect between sensory and emotional disturbances associated with nerve damage, emphasizing the importance of long‐term studies investigating how these affective disturbances change over time.

## Author contributions

R.H.M. wrote the manuscript and assisted in analysis and generation of figures, A.N. conceived and conducted the study, and performed the bulk of analysis the study, C.S. assisted during immunohistochemistry, T.P. provided technical assistance throughout and performed the hindpaw withdrawal tests, A.S.C. supervised the project. All authors have seen and commented on the finished manuscript.

## Supporting information


**Table S1.** Major domains of good laboratory practice to minimize experimental bias.
**Table S2.** Number of individual sections analysed per animal (*n* = 9/group), illustrating the technical replicates employed during c‐Fos analysis.
**Table S3.** Summary of c‐Fos immunoreactivity within the subnuclei and along the rostro‐caudal gradient of the central amygdala. SNT resulted in significantly higher densities of immunoreactivity across all levels and nuclei compared to naive. Data were analysed using three‐way ANOVA and Holm–Sidak *post hoc* multiple comparisons. *Denotes significant difference compared to naive, ^†^Denotes significant difference compared to sham – both *p* < 0.05.
**Data S1.** Supplementary results.
