# Supplementary material for: Enhanced c‐Fos expression in the central amygdala correlates with increased thigmotaxis in rats with peripheral nerve injury
Source: Eur J Pain. 2016 Mar 31;20(7):1140–54. doi: 10.1002/ejp.839 (PMC4950342; doi:10.1002/ejp.839)
Supplement: Supplementary file 1 [file EJP-20-1140-s001.odt]

Supplementary Materials

Table S1: Major domains of good laboratory practice to minimize experimental bias.

| Characteristic | Description |
| --- | --- |
| Sample Size calculation | Group size was determined by sample size estimation by SigmaStat software v3.5 (ANOVA sample size, desired power = 0.8, α = 0.05). Effect size for estimation were derived from previous studied conducted in this group (Blackbeard et al., 2012). Details of sample size calculation for each experiment can be found in the Supplementary materials. |
| Inclusion and Exclusion criteria | In experiments looking at mechanical hypersensitivity, only rats that developed hind paw mechanical hypersensitivity of at least 30% change from baseline were included. |
| Randomisation | Animals were allocated to experimental group by picking numbers out of a hat. |
| Allocation Concealment | The experimenter conducting open field also performed the SNT surgery, so was aware of group allocation. Cage labels were masked prior to behavioural tests to reduce this effect, however as pharmacological interventions were not investigated, robust allocation concealment is not crucial. |
| Reporting of animals excluded from analysis | Experimental exclusion criteria included the 30% reduction in mechanical hypersensitivity mentioned above, in addition to situations involving breach of experimental conditions. Additionally, humane endpoints were defined as follows: Any rat with hunched posture, marked behavioural change, exudates around wound, or sensitivity to palpitation on handling attributable to surgery, infection resulting from surgery or otherwise was excluded. Any rat with significant surgical complications or whose general health deteriorated was excluded. Very rarely, limited motor dysfunction or autotomy may occur after SNT surgery. If a persistent motor impairment prevents the animal from reaching food and/or water, the animal was excluded. Full details of all exclusions are given at the start of the results section. |
| Blinded measurement, assessment, and analysis of outcome | An independent experimenter assigned codes to the open field video files and histological slides prior to analysis. These codes were not broken until analysis was complete. SNT injured rats show hind limb posture changes compared to sham or naïve animals, making allocation concealment difficult to maintain, however measures to maintain blinding were still applied. |

Table S2: Number of individual sections analysed per animal (n=9/group), illustrating the technical replicates employed during c-Fos analysis.

|  | Mean | Min | Max |
| --- | --- | --- | --- |
| Naïve | 3.50 | 3 | 4 |
| Sham | 3.56 | 2 | 5 |
| SNT | 3.58 | 2 | 5 |

Table S3: Summary of c-Fos immunoreactivity within the subnuclei and along the rostro-caudal gradient of the central amygdala. SNT resulted in significantly higher densities of immunoreactivity across all levels and nuclei compared to naïve. Data was analysed using 3-Way ANOVA, and Holm-Sidak post-hoc multiple comparisons. * denotes significant difference compared to naïve, † denotes significant difference compared to sham – both p<0.05

|  | cells/mm2 | Naive | Sham | SNT |
| --- | --- | --- | --- | --- |
| Subnuclei | CeM | 3.97 SEM 0.95 | 6.19 SEM 1.46 | 15.77 SEM 1.61 * |
| CeC | 11.26 SEM 1.98 | 18.08 SEM 2.92 | 28.61 SEM 2.19 *† |
| CeL | 13.60 SEM 2.28 | 21.82 SEM 6.19 | 36.94 SEM 5.15 *† |
| Level | Rostral | 8.93 SEM 5.98 | 11.97 SEM 2.46 | 24.03 SEM 1.44 *† |
| Intermediate | 6.59 SEM 1.07 | 11.16 SEM 2.42 | 20.00 SEM 2.24 *† |
| Caudal | 21.56 SEM 4.13 | 37.01 SEM 8.02 | 54.05 SEM 8.01 *† |
| Subnuclei * Level | CeM – rostral | 7.73 SEM 2.28 | 11.13 SEM 2.56 | 24.67 SEM 2.63 |
| CeM - intermediate | 3.71 SEM 0.98 | 4.97 SEM 1.12 | 14.87 SEM 1.96 |
| CeM - caudal | 3.28 SEM 0.82 | 5.54 SEM 1.46 | 10.64 SEM 2.10 |
| CeC - rostral | 13.11 SEM 3.04 | 17.33 SEM 3.28 | 27.97 SEM 1.72 |
| CeC - intermediate | 7.52 SEM 1.40 | 14.32 SEM 2.66 | 21.78 SEM 2.97 |
| CeC - caudal | 23.46 SEM 6.37 | 35.32 SEM 7.95 | 49.24 SEM 4.96 |
| CeL - rostral | 8.96 SEM 1.71 | 15.23 SEM 3.97 | 26.15 SEM 3.14 |
| CeL - intermediate | 14.14 SEM 2.51 | 23.49 SEM 9.93 | 35.32 SEM 6.69 |
| CeL - caudal | 23.50 SEM 2.72 | 36.92 SEM 13.60 | 72.28 SEM 16.33 |

# Supplementary Results

Extra detail on non-significant findings

## Immunoreactivity

- Naïve animals showed no significant differences between rostro-caudal levels (caudal vs. intermediate p=0.098, caudal vs. rostral p=0.19, and rostral vs. intermediate p=0.74).

- No evidence of lateralisation was observed following open field exposure, with c-Fos immunoreactivity density being similar across hemispheres and all experimental groups (2-way RM ANOVA Ce: p=0.87, CeM p=0.78, CeL p=0.82, CeC p=0.45).

- Holm-Sidak post-hoc multiple comparisons within groups revealed no differences between subnuclei in naïve animals (CeM vs CeC p=0.03, CeM vs CeL p=0.03, CeC vs CeL p=1). 

## Correlations

- Minimal correlations were observed with hind paw withdrawal thresholds (PWT) and other outcomes, with a negative correlation between baseline PWT and faecal boli observed in naïve animals, with sham animals exhibiting a negative correlation between distance travelled in the inner zone and baseline PWT. No correlations were seen with hind paw withdrawal in the SNT group.

- Correlations between open field behaviour and c-Fos immunoreactivity in the Ce were minimal in naïve (duration and CeL only), and sham (no significant correlations) as compared to SNT.

## Power Calculation (a priori)

- Group sizes of 5 (hind paw withdrawal) and 8 (thigmotaxis) were determined by sample size calculations (SigmaStat, version 3.5; ANOVA sample size, desired power=0.8, α=0.05), with effect sizes for estimation derived from previous studies looking at the effect of spinal nerve transection on open field behaviour (Blackbeard et al., 2012).

- The data used is shown in the following two tables (POD, post-operative day):

| Thigmotaxis/Inner Zone Activity (POD7) | Naive | SNT |
| --- | --- | --- |
| Mean | 14.4 | 5.4 |
| SEM | 2 | 3.7 |

| Ipsilateral Hindpaw Withdrawal Thresholds | Naive | SNT |
| --- | --- | --- |
| Mean (Baseline/POD7) | 49.4 / 49.4 | 52.9 / 33 |
| SEM (Baseline/POD7) | 1.7 / 1.7 | 1.3 / 3.1 |

Post-Hoc power calculations on secondary outcome measures

The study was powered to detect differences in the primary outcome measure of thigmotaxis (frequency of entry to the inner zone) and not the behaviours described by the reviewer. Post-hoc power calculations have been performed to check whether the lack of significance seen is a result of a lack of power.

Using the below data, rearing was calculated to have a power of 1- β = 0.61.

Table S4: Rearing Power Calculation data

| Sample Size | 9 |
| --- | --- |
| Naïve Mean | 84.1 |
| SNT Mean | 61.33 |
| Standard Deviation | 21.6 |

Distance travelled was found to be adequately powered (1- β = 0.99).

Table S5: Distance Travelled Power Calculation Data

| Sample Size | 9 |
| --- | --- |
| Naïve Mean | 7505.42 |
| SNT Mean | 6342.27 |
| Standard Deviation | 427.23 |

#
